# Supplementary material for: A Likelihood Approach for Real-Time Calibration of Stochastic Compartmental Epidemic Models
Source: PLoS Comput Biol. 2017 Jan 17;13(1):e1005257. doi: 10.1371/journal.pcbi.1005257 (PMC5240920; doi:10.1371/journal.pcbi.1005257)
Supplement: S1 File — (TAR.GZ) [file pcbi.1005257.s014.tar.gz › HSPH_Online-SI-Revision/PF08/n1-extreme/n1-extreme_PF_table8.pdf]

1	R0	{2.95019, 14.2854}	{2.32942, 15.5102}	{{1.70379, 9.95941}, {2.88771, 20.}}	{21.3795, 23.5661}	inf	19.	48.3642	{12.4298, 86.7728}	168.929	0.404408	newly	10.	18.32	{6., 34.}	99.6	0.279989	Reff	0.253716	0.429817	{0.258274, 0.622884}	70.581	ar	924.	972.56	{822., 1066.}	11.71
2	R0	{2.67565, 16.946}	{2.01269, 14.5091}	{{1.38421, 8.47024}, {2.77039, 19.5051}}	{26.5201, 20.6224}	inf	31.	33.9062	{7.38534, 63.0467}	55.707	0.26725	newly	14.	14.53	{4., 27.}	57.3571	0.295121	Reff	0.232782	0.558484	{0.332957, 0.834911}	140.031	ar	926.	886.61	{648., 1028.}	11.7052
3	R0	{2.40066, 16.6543}	{2.01341, 15.1444}	{{1.32708, 9.73064}, {2.83768, 19.6283}}	{22.9494, 18.6508}	inf	69.	52.4199	{17.3672, 105.07}	47.3359	0.293967	newly	26.	21.52	{9., 39.}	44.0769	0.245529	Reff	0.249669	0.597436	{0.333367, 0.897604}	139.284	ar	916.	888.06	{658., 1043.}	12.7402
4	R0	{1.9294, 15.4361}	{1.94183, 15.19}	{{1.26168, 8.9663}, {2.58943, 20.}}	{22.1337, 21.8214}	inf	64.	41.0733	{9.57164, 83.8905}	50.0723	0.362697	newly	26.	16.32	{5., 32.}	51.	0.356937	Reff	0.507432	0.614097	{0.335478, 0.895382}	38.0378	ar	796.	855.89	{615., 1039.}	18.4611
5	R0	{2.92859, 19.5393}	{1.69517, 15.7293}	{{1.23802, 9.07453}, {2.13551, 20.}}	{42.1166, 20.6721}	inf	46.	21.1879	{3.57539, 45.811}	59.5669	0.530018	newly	14.	7.89	{1., 15.}	52.7857	0.452681	Reff	0.251859	0.554965	{0.337535, 0.759592}	120.434	ar	927.	781.22	{528., 976.}	17.8382
6	R0	{1.79622, 12.3148}	{1.85948, 14.6308}	{{1.28574, 8.96131}, {2.59483, 19.8873}}	{22.2708, 33.0921}	inf	28.	34.54	{7.00419, 72.0729}	64.0115	0.268232	newly	16.	15.1	{3., 29.}	49.75	0.285846	Reff	0.555032	0.724063	{0.430157, 1.00586}	40.2856	ar	734.	809.05	{585., 1001.}	20.9142
7	R0	{2.45855, 17.1776}	{2.04873, 14.3173}	{{1.4182, 6.90165}, {2.77846, 20.}}	{22.147, 23.6979}	inf	47.	39.5721	{11.1492, 69.778}	47.431	0.281781	newly	18.	16.89	{6., 30.}	47.6111	0.255207	Reff	0.358948	0.643558	{0.426845, 0.88524}	79.6486	ar	871.	850.37	{611., 1016.}	14.2606
8	R0	{2.20009, 15.519}	{1.84365, 15.2001}	{{1.3056, 8.97135}, {2.54381, 20.}}	{23.3385, 23.8263}	inf	24.	24.3106	{2.71527, 46.2222}	64.7843	0.401419	newly	12.	9.33	{1., 20.}	53.4167	0.431336	Reff	0.378415	0.531636	{0.283072, 0.806756}	51.4497	ar	839.	837.24	{584., 1023.}	16.2336
9	R0	{2.06493, 9.6414}	{2.22836, 14.9435}	{{1.58693, 9.75913}, {2.98375, 19.2893}}	{20.5465, 60.1938}	inf	11.	52.6043	{15.5445, 93.4806}	380.781	0.595236	newly	7.	21.31	{8., 36.}	206.429	0.421451	Reff	0.489388	0.53225	{0.296928, 0.801344}	30.1915	ar	777.	934.25	{750., 1047.}	22.9151
10	R0	{2.60775, 19.6936}	{1.74356, 14.8404}	{{1.30596, 7.70515}, {2.34847, 20.}}	{34.3148, 25.2555}	inf	57.	23.7941	{2.15879, 52.0566}	63.8881	0.65223	newly	14.	9.45	{1., 23.}	59.3571	0.509172	Reff	0.341615	0.564031	{0.312603, 0.794759}	71.1647	ar	889.	791.56	{586., 969.}	16.4297
11	R0	{1.99168, 8.13555}	{2.16426, 14.8102}	{{1.54189, 9.11726}, {2.90446, 20.}}	{21.8313, 83.6697}	inf	7.	38.519	{10.161, 71.5543}	452.027	0.63924	newly	6.	15.54	{5., 25.}	166.	0.355695	Reff	0.478003	0.552959	{0.354461, 0.76853}	30.0145	ar	772.	908.19	{722., 1044.}	20.6878
12	R0	{1.79304, 9.18941}	{2.11156, 14.9147}	{{1.5278, 9.87245}, {2.76283, 20.}}	{24.8367, 66.7275}	inf	15.	52.1102	{15.0978, 88.6862}	255.123	0.509352	newly	9.	21.64	{9., 34.}	147.556	0.379938	Reff	0.505637	0.584418	{0.354096, 0.858201}	32.4695	ar	725.	912.08	{707., 1033.}	28.3503
13	R0	{1.84016, 18.3431}	{1.66348, 14.9572}	{{1.22238, 8.29463}, {2.2747, 20.}}	{21.1192, 22.6898}	inf	80.	22.5204	{2.0917, 43.8319}	71.9987	0.78354	newly	33.	8.68	{1., 18.}	73.697	0.824406	Reff	0.651417	0.594265	{0.335297, 0.830998}	23.1622	ar	762.	758.87	{485., 974.}	19.0722
14	R0	{1.94221, 9.85971}	{2.08441, 16.0779}	{{1.45892, 7.55085}, {2.96488, 20.}}	{21.8138, 57.0696}	inf	4.	32.0609	{4.56827, 66.9014}	707.472	0.756071	newly	2.	12.4	{3., 25.}	522.	0.669197	Reff	0.388442	0.521238	{0.263223, 0.783324}	47.0764	ar	805.	896.55	{681., 1039.}	18.4981
15	R0	{1.78578, 12.0541}	{1.73874, 15.4898}	{{1.23562, 8.31964}, {2.34835, 20.}}	{19.9972, 39.3721}	inf	20.	24.3378	{1.89582, 59.5389}	78.4598	0.430247	newly	12.	8.95	{1., 21.}	57.25	0.476874	Reff	0.494661	0.55966	{0.302027, 0.789405}	33.3659	ar	732.	786.67	{527., 990.}	21.6052
16	R0	{1.94463, 16.5603}	{1.86511, 13.6568}	{{1.24271, 7.10524}, {2.5898, 18.4249}}	{20.623, 22.3623}	inf	49.	27.8696	{2.03239, 59.5502}	57.5851	0.558499	newly	20.	11.68	{1., 25.}	56.9	0.514238	Reff	0.536718	0.550537	{0.284998, 0.798343}	29.8789	ar	757.	696.8	{393., 894.}	18.7081
17	R0	{2.08901, 16.4314}	{1.76321, 15.1084}	{{1.24295, 7.95085}, {2.39659, 20.}}	{22.2816, 21.3602}	inf	74.	38.9787	{7.03977, 77.4168}	53.9071	0.466172	newly	26.	15.63	{3., 30.}	51.0385	0.402034	Reff	0.48465	0.607664	{0.333277, 0.893749}	40.3902	ar	826.	806.	{558., 1011.}	17.3075
18	R0	{2.76419, 18.56}	{2.0741, 15.0688}	{{1.49619, 9.56367}, {2.79774, 20.}}	{26.2698, 21.9219}	inf	52.	48.1851	{12.2842, 81.5807}	46.5428	0.269712	newly	13.	19.22	{6., 33.}	73.6923	0.24953	Reff	0.243249	0.553327	{0.327237, 0.796795}	127.474	ar	920.	902.04	{689., 1036.}	11.3326
19	R0	{2.3821, 17.1399}	{2.09091, 15.3394}	{{1.54754, 10.6403}, {2.75447, 20.}}	{18.6371, 18.1379}	inf	44.	53.8093	{14.9091, 85.3788}	58.503	0.248492	newly	11.	21.57	{8., 34.}	107.909	0.295898	Reff	0.32873	0.630215	{0.422335, 0.84753}	91.9017	ar	883.	887.02	{721., 1018.}	10.7271
20	R0	{2.78314, 19.6127}	{1.84039, 14.4834}	{{1.30838, 7.80655}, {2.41556, 20.}}	{34.0296, 26.9743}	inf	20.	19.5786	{2.5461, 43.5878}	65.8019	0.386022	newly	5.	7.85	{1., 17.}	93.8	0.385755	Reff	0.18647	0.518217	{0.282248, 0.756436}	177.909	ar	943.	834.1	{580., 1023.}	15.8918
21	R0	{1.85124, 14.6794}	{1.71345, 14.641}	{{1.23681, 8.97269}, {2.2972, 19.8945}}	{18.831, 22.2763}	inf	67.	38.6965	{5.48204, 69.551}	55.9841	0.460952	newly	31.	14.83	{3., 30.}	58.1613	0.502283	Reff	0.596099	0.622601	{0.330206, 0.876829}	26.2909	ar	730.	789.22	{528., 1006.}	20.6795
22	R0	{2.1028, 14.9852}	{2.11516, 15.0834}	{{1.48619, 9.81941}, {2.71697, 20.}}	{19.0266, 20.5404}	inf	31.	35.3437	{8.11158, 73.8517}	61.5532	0.293674	newly	11.	14.19	{4., 28.}	67.9091	0.265443	Reff	0.395326	0.560007	{0.383358, 0.805692}	43.9403	ar	840.	898.47	{687., 1031.}	15.2155
23	R0	{1.86601, 10.2157}	{2.13635, 14.7642}	{{1.44163, 8.89126}, {2.75702, 19.5457}}	{24.0961, 51.2118}	inf	19.	48.6589	{13.7925, 108.359}	167.099	0.359322	newly	9.	21.05	{7., 43.}	145.222	0.333846	Reff	0.546741	0.634733	{0.427744, 0.92026}	30.0531	ar	747.	896.56	{657., 1019.}	24.1954
24	R0	{2.34032, 10.0992}	{2.27406, 14.8586}	{{1.57237, 9.50434}, {2.92457, 20.}}	{15.5598, 51.9421}	inf	19.	40.6086	{9.8687, 77.5156}	133.026	0.357202	newly	7.	15.67	{5., 29.}	136.714	0.359471	Reff	0.278498	0.442776	{0.250717, 0.676535}	62.7703	ar	887.	955.45	{764., 1048.}	13.9019
25	R0	{1.91548, 15.3401}	{1.89104, 14.5723}	{{1.27376, 9.34568}, {2.55029, 19.8472}}	{19.7363, 21.1977}	inf	36.	44.162	{11.6392, 87.6075}	62.8525	0.267233	newly	14.	18.12	{5., 35.}	63.5714	0.244459	Reff	0.599545	0.656625	{0.411656, 0.927657}	29.6961	ar	765.	837.17	{603., 1021.}	20.1373
26	R0	{2.75283, 10.6721}	{2.31956, 14.9889}	{{1.56932, 9.69214}, {3., 19.7368}}	{19.2484, 47.4751}	inf	11.	38.75	{9.01588, 70.144}	261.118	0.507443	newly	5.	15.18	{6., 27.}	210.4	0.445291	Reff	0.225732	0.43026	{0.252305, 0.657285}	90.8675	ar	926.	958.18	{761., 1060.}	10.8488
27	R0	{2.08367, 19.7849}	{1.73205, 14.5167}	{{1.27177, 8.70685}, {2.3259, 20.}}	{22.1779, 26.8882}	inf	70.	27.4604	{2.90309, 61.2854}	64.9651	0.640946	newly	16.	10.63	{1., 22.}	56.6875	0.459527	Reff	0.43132	0.594604	{0.366401, 0.79559}	44.1922	ar	833.	802.88	{569., 983.}	15.3205
28	R0	{2.64334, 17.1765}	{2.11646, 15.4116}	{{1.41122, 7.32446}, {2.91198, 20.}}	{23.5411, 19.9612}	inf	50.	41.0796	{9.11967, 75.7106}	51.2354	0.317495	newly	17.	16.15	{5., 28.}	48.7647	0.27078	Reff	0.227327	0.556916	{0.344537, 0.81034}	144.984	ar	934.	893.8	{656., 1046.}	11.955
29	R0	{2.87008, 13.3872}	{2.0089, 15.7994}	{{1.32865, 9.74614}, {2.6303, 20.}}	{30.6634, 29.8984}	inf	16.	33.4525	{8.34966, 56.428}	129.529	0.369888	newly	8.	12.67	{4., 22.}	82.375	0.288754	Reff	0.172205	0.465286	{0.241651, 0.714583}	170.259	ar	941.	884.38	{613., 1043.}	12.0298
30	R0	{2.06105, 19.6275}	{1.70571, 14.8435}	{{1.24523, 7.92712}, {2.29733, 20.}}	{22.9596, 24.8215}	inf	18.	16.0673	{1.22944, 35.7899}	73.3698	0.532647	newly	6.	6.22	{0., 14.}	75.3333	0.49459	Reff	0.548239	0.567892	{0.332036, 0.789509}	26.7018	ar	747.	776.25	{528., 988.}	20.245
31	R0	{2.30764, 13.435}	{2.24515, 15.1139}	{{1.4802, 10.8601}, {2.9475, 19.7477}}	{19.3009, 24.5592}	inf	14.	44.82	{14.6339, 73.7293}	226.599	0.441206	newly	4.	18.4	{7., 31.}	363.	0.606648	Reff	0.313839	0.510941	{0.302786, 0.79158}	64.9699	ar	877.	935.64	{685., 1055.}	15.0832
32	R0	{2.30565, 11.9237}	{2.20251, 15.3488}	{{1.52678, 10.5822}, {2.92168, 20.}}	{17.7143, 35.1259}	inf	9.	45.7472	{11.7881, 83.6302}	413.263	0.628954	newly	6.	17.57	{7., 33.}	200.167	0.433315	Reff	0.341236	0.516095	{0.291576, 0.801751}	56.423	ar	853.	932.66	{736., 1059.}	15.6295
33	R0	{2.24031, 18.582}	{1.77844, 15.6736}	{{1.27426, 9.78558}, {2.36011, 20.}}	{25.9488, 19.5275}	inf	83.	37.8922	{7.01171, 77.9736}	58.1787	0.514711	newly	29.	14.45	{2., 26.}	54.3103	0.457649	Reff	0.396535	0.577608	{0.308856, 0.847952}	55.6727	ar	864.	804.36	{574., 1000.}	15.294
34	R0	{2.33306, 15.9759}	{2.06607, 15.0091}	{{1.4917, 10.366}, {2.73573, 19.9052}}	{19.1478, 19.1057}	inf	34.	41.9812	{11.118, 73.9738}	58.6734	0.257273	newly	12.	17.01	{5., 30.}	67.25	0.234149	Reff	0.354625	0.57377	{0.367308, 0.825917}	62.9394	ar	854.	895.19	{706., 1036.}	14.2213
35	R0	{2.48357, 18.3573}	{2.02405, 14.9254}	{{1.37821, 8.0384}, {2.72516, 20.}}	{23.2178, 22.3899}	inf	55.	45.1621	{11.9356, 84.2544}	48.8755	0.299492	newly	20.	18.97	{6., 33.}	47.35	0.261288	Reff	0.442075	0.648973	{0.397527, 0.984689}	51.7159	ar	835.	866.6	{613., 1022.}	15.521
36	R0	{2.59548, 19.9046}	{2.00319, 14.9985}	{{1.33494, 8.966}, {2.76111, 20.}}	{25.6922, 24.7858}	inf	36.	44.2349	{6.53288, 85.9454}	71.855	0.325284	newly	9.	16.86	{4., 36.}	113.111	0.325256	Reff	0.399704	0.577407	{0.304792, 0.833684}	52.0542	ar	863.	875.21	{610., 1057.}	15.876
37	R0	{2.29641, 16.9868}	{1.70603, 15.0114}	{{1.21658, 8.18211}, {2.32007, 20.}}	{27.6738, 19.7075}	inf	41.	18.2576	{2.43759, 39.9187}	61.2032	0.56334	newly	12.	7.25	{1., 17.}	55.9167	0.503003	Reff	0.314608	0.554934	{0.298713, 0.758904}	79.5134	ar	877.	777.18	{514., 988.}	17.4139
38	R0	{2.65743, 15.4569}	{1.98023, 14.8696}	{{1.3562, 7.05533}, {2.82779, 20.}}	{27.9824, 22.8971}	inf	23.	37.8685	{4.18255, 79.9932}	100.521	0.360158	newly	9.	14.79	{4., 29.}	92.3333	0.300439	Reff	0.318892	0.574908	{0.322784, 0.842099}	83.3356	ar	897.	872.56	{597., 1038.}	14.6198
39	R0	{2.67953, 14.5132}	{2.06594, 14.8315}	{{1.39406, 10.4373}, {2.74624, 19.4201}}	{24.9412, 19.2894}	inf	24.	48.985	{14.6077, 89.852}	119.14	0.310869	newly	6.	21.19	{7., 41.}	255.833	0.467148	Reff	0.310825	0.580071	{0.340059, 0.944216}	87.8031	ar	894.	901.34	{695., 1048.}	13.3154
40	R0	{1.94594, 11.928}	{1.86868, 13.9886}	{{1.22638, 8.26611}, {2.46976, 19.0665}}	{19.6787, 32.8685}	inf	36.	21.5912	{1.60269, 51.5578}	64.4804	0.577373	newly	15.	8.89	{0., 21.}	60.4667	0.583043	Reff	0.447566	0.550251	{0.296293, 0.894455}	40.3637	ar	796.	689.11	{316., 884.}	19.5138
41	R0	{1.66297, 10.0047}	{1.60131, 14.7818}	{{1.18649, 7.01345}, {2.05549, 20.}}	{19.0307, 59.3019}	inf	25.	24.8271	{1.52837, 51.0126}	65.3229	0.421051	newly	8.	9.7	{1., 20.}	71.	0.380507	Reff	0.53215	0.623456	{0.389678, 0.811917}	30.0378	ar	716.	737.28	{518., 954.}	20.8911
42	R0	{2.91568, 16.4453}	{2.24868, 14.8783}	{{1.53569, 10.264}, {2.98923, 20.}}	{23.5451, 19.5076}	inf	25.	48.8608	{13.7089, 90.0596}	115.001	0.302885	newly	14.	19.66	{7., 38.}	70.7143	0.240197	Reff	0.23617	0.491403	{0.285216, 0.718578}	108.08	ar	919.	950.07	{750., 1060.}	10.8346
43	R0	{2.55898, 15.8889}	{1.9899, 14.7061}	{{1.39551, 7.15893}, {2.72524, 20.}}	{25.2499, 21.6732}	inf	46.	48.8909	{14.967, 89.0005}	54.9371	0.256732	newly	20.	20.5	{8., 36.}	48.2	0.241788	Reff	0.30196	0.635874	{0.367553, 0.887762}	110.667	ar	904.	868.88	{656., 1034.}	13.2345
44	R0	{2.28649, 15.9017}	{2.09443, 15.2404}	{{1.35978, 8.96268}, {2.82446, 20.}}	{20.8535, 19.2725}	inf	33.	38.2601	{8.30079, 78.1705}	68.3608	0.304082	newly	11.	15.36	{3., 29.}	79.2727	0.299292	Reff	0.324682	0.544611	{0.317856, 0.824952}	70.6713	ar	880.	898.81	{618., 1040.}	14.8193
45	R0	{2.65121, 18.6339}	{2.1592, 14.5387}	{{1.54501, 10.3121}, {2.77093, 19.2678}}	{20.8266, 23.3981}	inf	63.	48.6705	{15.8502, 80.5803}	46.084	0.290793	newly	16.	20.66	{8., 38.}	63.	0.237948	Reff	0.230655	0.552039	{0.359948, 0.801914}	139.335	ar	928.	911.61	{723., 1043.}	10.5636
46	R0	{2.52291, 18.0251}	{2.03229, 15.5291}	{{1.45467, 9.76557}, {2.77572, 20.}}	{23.0976, 18.5709}	inf	48.	46.2725	{10.7575, 96.9457}	55.1757	0.296292	newly	15.	18.23	{6., 33.}	59.2667	0.249822	Reff	0.32041	0.564653	{0.34222, 0.804203}	77.4847	ar	894.	893.28	{676., 1039.}	12.8479
47	R0	{2.22618, 17.1225}	{2.08018, 15.1965}	{{1.37251, 9.73678}, {2.84217, 20.}}	{19.772, 19.7977}	inf	64.	51.975	{12.2841, 108.471}	51.4131	0.315259	newly	23.	20.65	{8., 39.}	46.0435	0.24574	Reff	0.454141	0.630089	{0.342857, 0.983011}	47.3618	ar	850.	887.03	{630., 1047.}	16.6671
48	R0	{2.04405, 16.073}	{1.91901, 14.7237}	{{1.21645, 9.72446}, {2.59806, 19.8463}}	{20.9485, 20.202}	inf	45.	45.3268	{12.1453, 102.831}	65.7951	0.303428	newly	18.	19.46	{6., 37.}	58.3333	0.259245	Reff	0.533497	0.760717	{0.476774, 1.09749}	47.2442	ar	779.	811.78	{524., 1010.}	20.8293
49	R0	{2.84396, 18.097}	{2.26766, 14.7214}	{{1.53123, 8.01937}, {2.89662, 19.9024}}	{21.4825, 21.781}	inf	41.	38.4313	{10.9964, 71.3461}	47.0465	0.253637	newly	16.	15.83	{6., 25.}	41.6875	0.198191	Reff	0.170638	0.475898	{0.29679, 0.746451}	178.894	ar	952.	927.27	{696., 1044.}	10.9779
50	R0	{1.85341, 18.0975}	{1.8598, 14.0398}	{{1.24719, 8.42466}, {2.46976, 18.8445}}	{21.6527, 24.2483}	inf	40.	24.0873	{1.29762, 51.992}	59.6435	0.529825	newly	17.	10.38	{1., 22.}	55.2941	0.495341	Reff	0.498567	0.551202	{0.292214, 0.861861}	36.1663	ar	754.	698.01	{403., 890.}	19.4629
